# The Sound of Silence: RNAi in Poly (ADP-Ribose) Research

**DOI:** 10.3390/genes3040779

**Published:** 2012-12-06

**Authors:** Christian Blenn, Philippe Wyrsch, Felix R. Althaus

**Affiliations:** Institute of Pharmacology and Toxicology, University of Zurich-Vetsuisse, Winterthurerstrasse 260, 8057 Zurich, Switzerland; E-Mails: christian.blenn@vetpharm.uzh.ch (C.B.); philippe.wyrsch@vetpharm.uzh.ch (P.W.)

**Keywords:** ADPR, PAR, PARP1, PARP2, PARG, siRNA

## Abstract

Poly(ADP-ribosyl)-ation is a nonprotein posttranslational modification of proteins and plays an integral part in cell physiology and pathology. The metabolism of poly(ADP-ribose) (PAR) is regulated by its synthesis by poly(ADP-ribose) polymerases (PARPs) and on the catabolic side by poly(ADP-ribose) glycohydrolase (PARG). PARPs convert NAD^+^ molecules into PAR chains that interact covalently or noncovalently with target proteins and thereby modify their structure and functions. PAR synthesis is activated when PARP1 and PARP2 bind to DNA breaks and these two enzymes account for almost all PAR formation after genotoxic stress. PARG cleaves PAR molecules into free PAR and finally ADP-ribose (ADPR) moieties, both acting as messengers in cellular stress signaling. In this review, we discuss the potential of RNAi to manipulate the levels of PARPs and PARG, and consequently those of PAR and ADPR, and compare the results with those obtained after genetic or chemical disruption.

## 1. The Life Cycle of Poly (ADP-Ribose)

**Figure 1:**
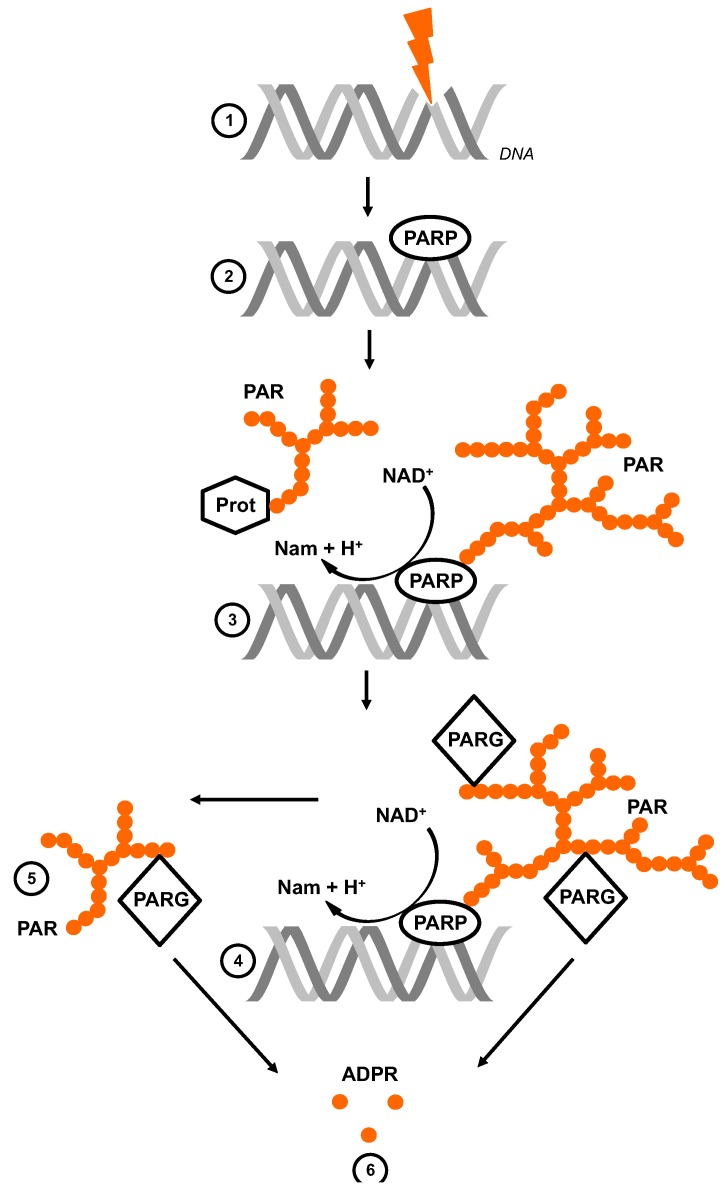
Poly(ADP-ribose) metabolism. Single-strand and double-strand breaks (SSB and DSB) in the DNA are induced by genotoxic stress (1). The nuclear PARPs bind to the damaged site and are thereby activated and produce ramified and highly negatively charged poly(ADPribose) (PAR) (2). They hydrolyze NAD^+^, releasing nicotinamide (Nam) and H^+^, catalyzing the successive transfer of ADPR to acceptors (Prot) (3). PARG is the catabolic enzyme with endo- and exoglycosidic activity (4). Its hydrolyzing activity leads to either free PAR chains (5) and/or monomeric ADPR (6).

Poly(ADP-ribosyl)-ation belongs to the nonprotein posttranslational modifications and is a metabolite of the enzymatic cofactor nicotinamide adenine dinucleotide (NAD^+^). Poly (ADP-ribose) polymerases (PARPs) cleave the nicotinic moiety from NAD^+^ and convert the ADP-ribose (ADPR) units into long APD-ribose polymers (PAR). At the protein level, the β-α-loop-β-α NAD^+^ fold is the most conserved region in PARPs and is therefore termed the PARP signature motif [1,2,3]. To date, 17 distinct PARP enzymes have been discovered and numbered accordingly. Recently a new nomenclature has been suggested that is based on their transferase activity (ARTD nomenclature [4]): PARP1 (ARTD1), PARP2 (ARTD2), PARP3 (ARTD3), PARP4 (ARTD4, vault-PARP), PARP5A (ARTD5, Tankyrase 1), PARP5B (ARTD6, Tankyrase 2), PARP6 (ARTD17), PARP7 (ARTD14, TIPARP, RM1), PARP8 (ARTD16), PARP9 (ARTD9, BAL1), PARP10 (ARTD10), PARP11 (ARTD11), PARP12 (ARTD12, ZH3HDC1), PARP13 (ARTD13, ZC3HAV1, ZAP1), PARP14 (ARTD8, BAL2 COAST6), PARP15 (ARTD7, BAL3), and PARP16 (ARTD15). However, not all PARP enzymes are proven poly(ADP-ribose) polymerases. Several of them seem to belong to the class of mono(ADP-ribosyl) transferases [5]. The nuclear PARP1 and PARP2 are the best characterized PARPs in mammals and are true poly(ADP-ribose) polymerases. PARP1 has a modular structure and starts its catalytic activity after binding to DNA nicks and breaks with the double zinc finger domain [2]. Recently, a third zinc finger-like structure was discovered. It is required for transmitting DNA-induced conformational changes to the catalytic domain [6,7]. The C-terminal catalytic domain sequentially transfers ADPR units from NAD^+^ to protein acceptors generating PAR. Third, in the automodification domain, specific glutamatic acid and lysine residues serve as acceptors of ADPR allowing the enzyme to poly(ADP-ribosyl)ate itself. While the level of PAR is very low under physiological condition, it rises 200-fold under genotoxic stress conditions. After DNA damage, PARP1 is rapidly recruited and its catalytic activity increases 10- to 500-fold to synthesize long and branched PAR chains (Figure 1) [3,8]. PARP1 is the main enzyme generating PAR and it is the main acceptor for covalent PAR modification. More than 90% of PAR originates from PARP1 during genotoxic stress [8,9]. PARP‑bound PAR can recruit many other proteins involved in distinct cellular functions, including enzymes involved in DNA repair [10,11,12,13,14,15,16]. The system of PAR accepting and/or interacting proteins is very complex and not completely understood. Moreover, novel proteome-wide analyses allow the identification of putative PAR binding proteins [10]. Automodification of PARP1 diminishes the affinity of the enzyme for DNA breaks. This provides a mechanism for removing PARP1 from damaged DNA and for local modulation of chromatin compaction and transcriptional regulation [11,17,18,19].

Apart from PARP1, PARP2 is activated by DNA strand breaks, as well [20,21]. The DNA-binding domain (DBD) of PARP2 is structurally different from that of PARP1 [20,22]. PARP2 binds less efficiently to DNA single-strand breaks (SSB) than PARP1 but it detects gap and flap structures [23]. PARP2 consumes less NAD^+^ compared to PARP1 due to its less efficient PAR synthesis [20]. It contributes only 5%–10% of the total PARP activity in response to DNA interruptions [20,24].

The enzymatic product of PARP1 and PARP2 activity PAR comprises a heterogeneous pool of negatively charged molecules that differ in length and branching. It is noteworthy that the hyperactivation of PARP1 and PARP2, due to genotoxic stress, consumes most of the NAD^+^ in a cell [25]. Free or protein-associated PAR, synthesized after a genotoxic insult, are rapidly degraded *in vivo* with a half-life of less than 40 sec, while the residual fraction is catabolized with a half-life of 6 min [26,27,28]. The degradation of PAR is catalyzed by PARG (PAR glycohydrolase), an enzyme with both endo- and exoglycosidic activities that hydrolyze the glycosidic bond between ADPR units. PARG produces primarily monomeric ADPR *via* exoglycosidic activity, albeit a few free PAR polymers may arise from endoglycosic cleavage (Figure 1) [29,30]. In addition, PARG displays less activity towards branched or short PAR (K_M_ ≈ 10 µM) compared with long and linear PAR molecules (K_M_ ≈ 0.1–0.4 µM) [29,30,31,32,33,34]. PARG is encoded by a single gene in mammals, and several splicing products are formed after transcription. They are translated into proteins of different molecular size, subcellular localization and the ability to cleave PAR. The nuclear mPARG-110/hPARG-111 isoform represents the full-length PARG protein in mice and humans and accounts for most of the PARG activity [35]. Recently another PAR-degrading enzyme has been described. The ADPR hydrolase 3 (ARH3) is structurally not related to PARG and is less efficient. Nevertheless, it provides PAR-degrading activity *in vitro* and in PAR-enriched mitochondria, as it has been demonstrated in a PARP overexpression system [36,37,38].

The two products of PARP/PARG interplay have been identified to exhibit different cellular signaling functions. The manipulation of either PARP or PARG activity modifies the occurrence of PAR and ADPR after genotoxic stress (Figure 1). This allows the study of distinct PAR and ADPR functions. Here, we discuss approaches to interfere with PAR metabolism to clarify the biological role of this nonprotein posttranslational modification (PAR) and its degradation product (ADPR).

## 2. Experimental Tools to Investigate PAR and ADPR

### 2.1. Chemical Inhibition of PAR Metabolizing Enzymes

**Figure 2:**
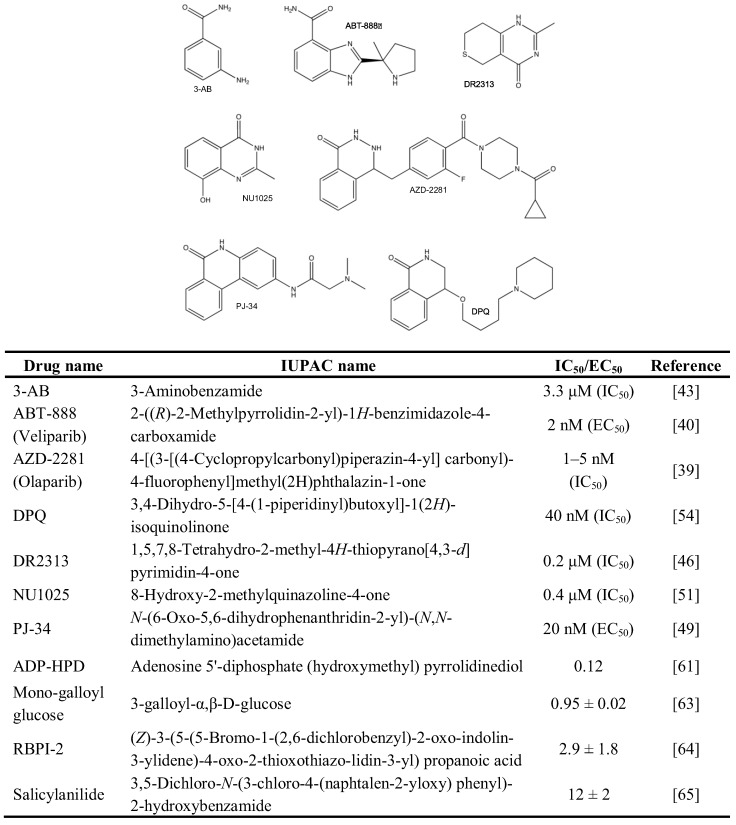
PARP inhibitors.

Within the last few decades, the concept of interfering with proteins involved in DNA repair and stress signaling has attracted a lot of attention in both basic and clinical research. To date, chemical inhibitors against PARP enzymes have reached the first level of clinical application. Almost all PARP inhibitors in preclinical and clinical studies compete with the substrate NAD^+^ for the catalytic domain leading to a reversible inhibition of enzyme activity. The third generation PARP inhibitors veliparib (ABT-888) and olaparib (AZD2281/KU-0059436) are the most clinically investigated, and their half-maximal inhibitory concentration (EC_50_ or K_i_) is in the nanomolar range for PARP1 and PARP2 (Figure 2) [39,40]. Both inhibitors are sufficiently bioavailable when administered orally and they are primarily used as anticancer drugs alone or in combination with other treatments (discussed in [41]). Moreover, the potential of PARP inhibitors to participate in the concept of synthetic lethality is under investigation [41,42].

The PARP inhibitors 3-Aminobenzamide (3-AB) [25,43,44,45], 1,5,7,8-Tetrahydro-2-methyl-4*H*-thiopyrano[4,3-*d*]pyrimidin-4-one (DR2313) [25,46], *N*-(6-Oxo-5,6-dihydrophenanthridin-2-yl)-(*N*,*N*-dimethylamino)acetamide (PJ-34) [25,47,48,49,50], 8-Hydroxy-2-methylquinazoline-4-one (NU1025) [51,52,53], and 3,4-Dihydro-5-[4-(1-piperidinyl)butoxyl]-1(2*H*)-isoquinolinone (DPQ) [54,55,56,57], were extensively used to suppress PARP activity *in vitro*, in cells and animals (Figure 2). They share the potential to interfere with both PAR-synthesizing enzymes activated by DNA damage due to the homology in the catalytic domain of PARP1 and PARP2, albeit with different kinetics. To overcome this problem, Moroni and co-workers developed a set of specific PARP2 inhibitors [58]. Beside synthetic PARP inhibitors, a panel of naturally occurring molecules was discovered that have PARP-suppressing activity. These compounds cover such different chemical structures as tryptophan derivatives, purines, xanthins, vitamins, hormones and metals [59].

**Figure 3:**
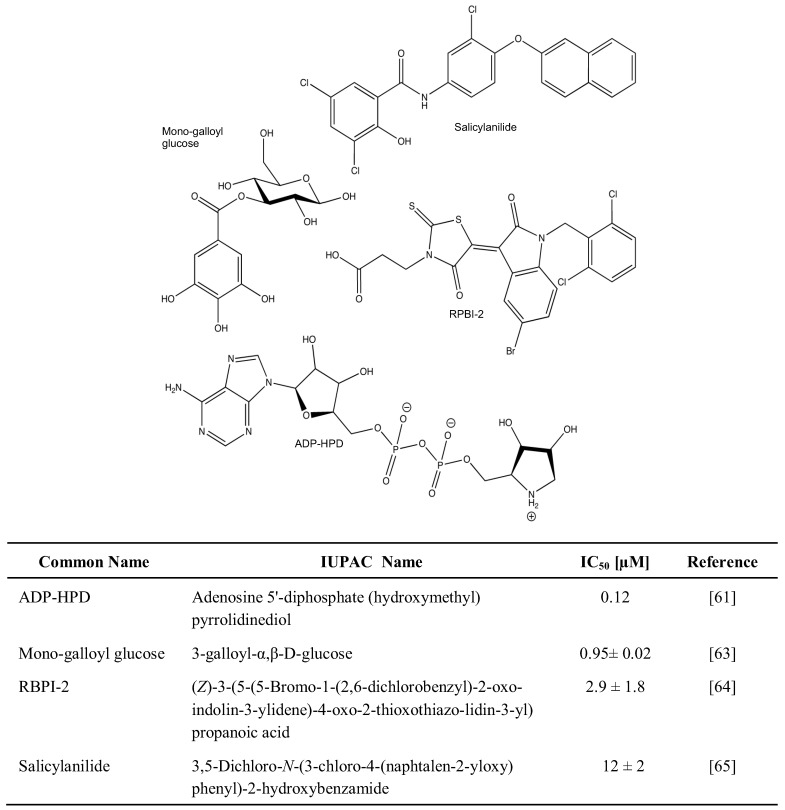
PARG inhibitors.

Much less is known about the inhibitors of PARG, as was recently reviewed [60]. Whereas a number of natural and synthetic molecules have been described to exhibit PARG-suppressing activity, most of them are restricted in terms of bioavailability and/or specificity. Nevertheless, the use of Adenosine 5'-diphosphate (hydroxymethyl) pyrrolidinediol (ADP-HPD) [61,62], 3-Galloyl-α,β-D-glucose [63], (*Z*)-3-(5-(5-Bromo-1-(2,6-dichlorobenzyl)-2-oxoindolin-3-ylidene)-4-oxo-2-thioxo-thiazolidin-3-yl) propanoic acid [64], and 3,5-Dichloro-*N*-(3-chloro-4-(naphtalen-2-yloxy)phenyl)-2-hydroxy benzamide [65] may inhibit PARG specifically and help understand its role within a wider biological context (Figure 3).

### 2.2. Genetic Disruption of PARPs and PARG

In the mid-1990s, the first PARP knock out (k.o.) mice were created to provide more detailed and specific information about the physiological role of PARP enzymes in different cellular responses. Wang *et al.* generated in 1995 a PARP1 k.o. mouse line by interrupting exon 2 [66]. The existence of these mice suggested initially that PARP1 is dispensable during embryogenesis. These mice were fertile, obviously healthy and revealed no dramatic phenotype. However, isolated cells derived from *Parp1^−/−^* mice had a marginally lower proliferation rate unrelated to DNA damage. Nevertheless, there was an unusual and unexpected development of skin hyperplasia in around 1/3 of mice related to progressive aging. The observed skin abnormalities included thickening of all layers of the epidermis, active proliferation of keratinocytes, development of intracellular edema and an inflammatory response [66]. As 70% of these mice remained free of any skin problems, a correlation between this phenotype and the absence of PARP1 seemed unlikely. Ménissier de Murcia *et al.* developed a second *Parp1^−/−^* mouse strain using a different ablation strategy by interrupting exon 4 [67], which was completely free of any skin pathologies as discussed in more detail by Rhun *et al.* [68]. These PARP1 k.o. mice exhibited an extreme sensitivity to whole body γ‑radiation, indicating a functional link between PARP1 and DNA stress. This first report was later confirmed by Wang *et al.* investigating their first *Parp1^−/−^* mouse type [69]. Additionally, Ménissier de Murcia and colleagues showed that the whole body exposure to 8 Gy resulted in a very short half-life of four days with a complete lethality after eight days, compared to 50% lethality observed in control mice (14–20 days). Dramatic lethality with extensive necrosis occurred in the epithelial lining of the small intestine [67]. Furthermore, high sensitivity to intra-peritoneal administration of 75 mg/kg of body weight of the alkylating agent *N*-methyl-*N*-nitrosourea (MNU) was observed. They determined 80% lethality within the first week in *Parp1^−/−^* mice compared to 40% for control mice until week eight.

Results obtained in these two different *Parp1^−/−^* mice lines demonstrated the involvement of PARP1 in processing genotoxic attacks. Both authors concluded that the lack of the *Parp1* gene led to an impaired DNA repair capacity, an accumulation of DNA strand breaks and, subsequently, high genomic instability. The results of the k.o. experiments performed by Wang *et al.* and Ménissier de Murcia *et al.* are summarized in Table 1. None of the approaches to diminish the *Parp1* gene resulted in the expression of fully functional truncates. However, Wang *et al.* reported a truncated 1.2 kb transcript generating a PARP1 fragment but without any enzymatic activity.

In 1999, a partial functional redundancy between PARP1 and PARP2 was observed by Amé *et al.* [20]. As a logical consequence, mice lacking PARP2 were needed. Therefore, Ménissier de Murcia and co-workers generated the first *Parp2^−/−^* mice line by disrupting exon 9 [70]. These manipulated mice were fertile and viable like the *Parp1^−/−^* mice. Interestingly, they showed an even higher sensitivity to genotoxic challenges than their *Parp1^−/−^* counterparts. Moreover, *Parp2^−/−^* mice displayed abnormalities in spermiogenesis caused by delayed nuclear elongation. They developed lipodystrophy characterized by adipodegeneration, as well as derailed differentiation of preadipocytes to adipocytes. It was also reported that genetic depletion of *Parp2^−/−^* resulted in reduced CD4^+^CD8^+^ thymocyte cellularity, the overexpression of the proapoptotic proteins Noxa and Puma as well as a reduced expression of the T-cell receptor (TCR) α [21,71,72,73,74]. The importance of the PAR metabolizing enzymes PARP1 and PARP2 is further illustrated by the fact that a double k.o. results in embryonic death. This can be observed already in the early stage of gastrulation leading to a complete loss of the offspring [70,75].

After the first demonstration of PARP1 and PARP2 functions using k.o. mice, another breakthrough was made by the disruption of the PAR degrading enzyme PARG. First, Koh and colleagues targeted exon 4 within the *parg* gene [76]. This led to early peri-implantation lethality resulting in the failure of the embryos to progress and the subsequent degeneration of the blastocyst. However, embryonic trophoblast stem cell lines established from early PARG null embryos could be kept viable in culture, but only when co-cultured with the traditional PARP inhibitor benzamide (0.5 mM). Moreover, the Parg^−/−^ cells were characterized by reduced growth, an accumulation of PAR and an increased sensitivity to the alkylating agent *N*-methyl-*N’*-nitro-*N*-nitrosoguanidine (MNNG, 20 and 100 μM). An improved strategy to impair *Parg* gene in mice to overcome lethality problems was reported by Cortes *et al.* [77]. They targeted exons 2 and 3 resulting in the depletion of only PARG_110_ protein isoform normally found in the cell nucleus. Due to this elegant approach the mice were viable, fertile and phenotypically normal but hypersensitive to alkylating agents (MNU, 150 mg/kg of body weight) and ionizing radiation (10 Gy of whole body γ-radiation). However, Meyer-Ficca *et al.* reported a reduced number of litters as well as a decreased number of pups per litter in *Parg^Δ2−3^*mice [78]. Moreover, the *Parg^Δ2−3^* mice were prone to septic shock induced by lipopolysaccharides (LPS, 30 mg/kg of body weight). Both PARG manipulated mouse models are presented in Table 1. 

### 2.3. RNA Interference

Sense RNAs have been typically introduced as negative specificity controls using RNAs synthesized *in vitro* in antisense studies in the 1990s [79]. Interestingly, Guo and Kemphues found that control sense as well as antisense RNA molecules resulted in similar phenocopies when administered to the worm *Caenorhabditis elegans* [80]. Further studies in *C. elegans* revealed that the interfering RNA could be transported from the site of initial delivery to most cells and tissues in the worm and this systemic response was termed RNA interference or RNAi [81]. Subsequently, it was discovered that double-stranded RNA (dsRNA) rather than single-stranded antisense RNA (ssRNA) was responsible for the sequence specific degradation of targeted endogenous mRNA in *C. elegans* [82]. This form of posttranscriptional gene silencing in response to transgene sequences was previously demonstrated in plants and also in fungi representing an evolutionary well-conserved mechanism. Napoli *et al.* introduced a transgene designed to overexpress chalcone synthase putatively leading to an increase in purple pigment production in petunia flowers. Surprisingly, more than 40% of the transgenic plants developed white or variegated flowers rather than purple [83]. A similar phenomenon of target gene repression in a different model organism was discovered in experiments using the fungus *Neurosporus crassa* [84].

**Table 1:** Murine phenotypes of genetic PARP or PARG disruption.

Deletion	Phenotype		Ref.
*Parp1*	◆	Accumulation of DNA strand breaks and impaired DNA repair	[66,67,68]
	◆	High genomic instability	
	◆	Hypersensitivity to γ-irradiation and alkylating agents	
	◆	No defects in viability, fertility, development or tissue differentiation	
*Parp2*	◆	High genomic instability	[70,73]
	◆	Hypersensitivity to γ-irradiation and alkylating agents	
	◆	Impaired adipogenesis, thymopoiesis, and spermatogenesis	
	◆	No defects in viability, fertility, development or tissue differentiation	
*Parp1, Parp2*	◆	Embryonic lethality at onset of gastrulation	[70,75]
*Parg*	◆	Peri-implantation lethality	[76]
*Parg* *^Δ2−3 ^*	◆	Increased responses to genotoxic treatment and septic shock	[77,78]
	◆	Phenotypically normal and viable	

**Figure 4:**
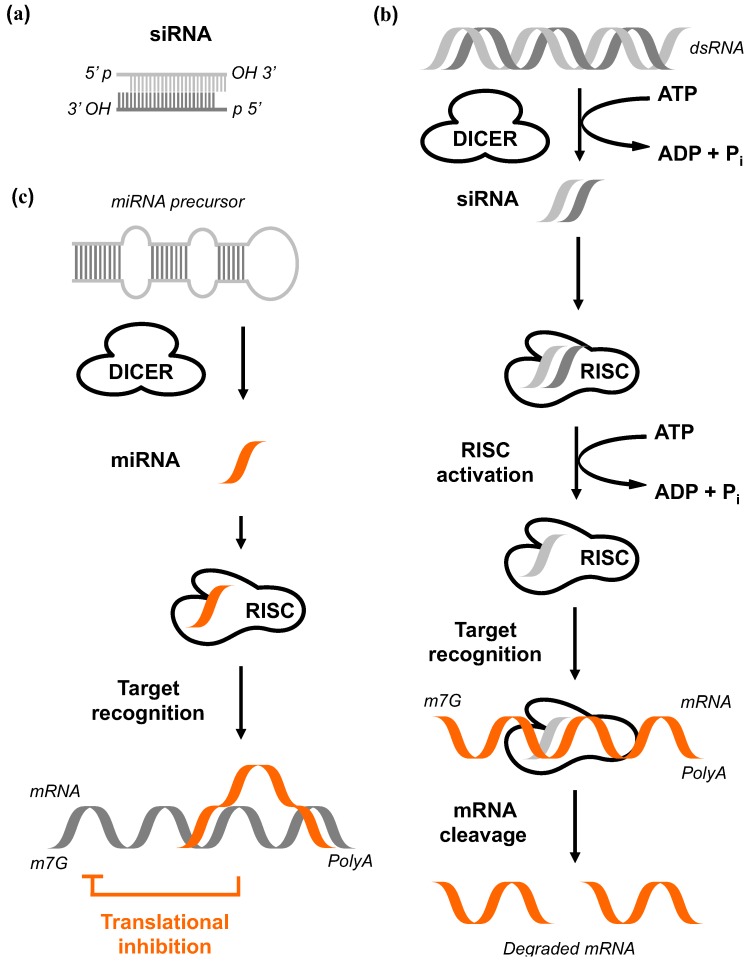
The pathway of RNA interference. **(a)** Highly specific siRNA which are 21–23nt double-stranded RNA (dsRNA) duplexes in size with symmetric 2-3 nucleotide 3’ overhangs and 5’ phosphate (P) and 3’ hydroxyl (OH) groups. **(b)** The dsRNA is cleaved by Dicer in an ATP-dependent reaction. The siRNAs are subsequently incorporated into a multicomponent RNA-inducing silencing complex (RISC). Activated RISC unwinds the siRNA in an ATP-dependent fashion. The resulting antisense strand guides the RISC to its complementary mRNA before endonucleolytic cleavage of target mRNA. The free ends of the mRNA fragments are rapidly degraded by cytoplasmic nucleases. This ultimately results in the loss of protein expression. **(c) ** Dicer cleaves the miRNA precursor to produce 22 nt miRNA. The single-stranded miRNAs are incorporated into RISC, followed by target recognition and final translational inhibition [104].

A first mechanistic model resolving this phenomenon originated from comprehensive biochemical and genetic studies on flies, worms, fungi, plants, and mammalian cells (Figure 4, reviewed by Hutvagner *et al.* [85]). The RNA-silencing response starts by processing the trigger dsRNA molecules into smaller fragments with 21 to 25 bp in size that are characterized by 3’ dinucleotide overhangs. The activity of an enzyme called Dicer is required for this process and it depends on adenosine triphosphate (ATP). This multidomain protein contains an ATP-dependent RNA helicase, a Piwi Argonaut and Zwille (PAZ) domain, two tandem RNase III domains, and a dsRNA-binding domain [86]. The final products of the Dicer activity were termed short interfering RNAs (siRNAi) [87]. Each siRNA binds subsequently to the RNA-induced silencing complex (RISC) that is composed of different protein subunits. In a next step, the siRNAs become unwound due to a helicase component of RISC. This allows base pairing between the antisense strand and the target mRNA [88]. Endonucleolytic cleavage of the target mRNA *via* the endonuclease Ago2 leads to fragments missing polyA tail or missing 5' 7-Methylguanosine cap leading subsequently to degradation [79]. The mechanistic overview is shown in Figure 4.

To date, four different types of small RNA molecules are described to possess RNA interference.

#### (I) Short interfering RNA (siRNA)

Synthetic siRNA was the first RNAi technology used in mammalian cells for sequence specific gene silencing [89]. The siRNA directly incorporates into RISC, where its guide strands binds and cleaves the target mRNA. After the release of cleaved mRNA, the guide-strand-bound RISC binds to another mRNA starting a new cleavage cycle. siRNAs are capable to cleave and subsequently suppress cytoplasmic or nuclear target mRNA [90]. 

#### (II) Short hairpin RNA (shRNA)

Short hairpin RNA (shRNA) has been developed as an alternative strategy to siRNA and was aimed for long-term gene silencing [91,92]. The shRNA is transcribed in the nucleus out of an expression vector bearing a short dsDNA sequence with a hairpin loop. The resulting transcript becomes further processed after coupling to the cytoplasmic RISC, following the identical fate as described above for siRNA. In practice, several aspects of shRNA differ intrinsically from siRNA reviewed by Rao *et al.* [93].

#### (III) MicroRNA (miRNA)

MicroRNAs (miRNAs) are highly conserved small noncoding RNAs, which play a role in regulating physiological and pathological cell functions. They are initially transcribed in the nucleus as primary transcripts from precursors generally located within either intergenic regions or introns of protein coding sequences [94]. After processing by an RNAse III endonuclease, the pre-miRNAs are exported to the cytoplasm and further cleaved by the Dicer producing 20–23 bp mature miRNAs. The miRNAs are subsequently loaded into RISC eliciting transcriptional inhibition with target mRNA degradation or sequestration in cytoplasmic P-bodies [95]. Compared to siRNAs and shRNAs, which require a perfect match with the target mRNA, miRNAs exert typically translational inhibition by binding to a partially complementary mRNA (Figure 4). Therefore, the change in the expression of a single miRNA may affect hundreds of different genes [96,97].

#### (IV) Bi-functional shRNA (bi-shRNA)

Bi-functional shRNAs (bi-shRNAs) were developed to exploit both the cleavage and translational inhibitions mechanisms of RNAi [93]. They consist of two stem-loop shRNAs structures. The cleavage-dependent unit perfectly matches passenger and guide-strand, whereas the cleavage-independent unit is composed of a mismatched double-strand. Both units are embedded in a miR-30 scaffold encoded in a plasmid vector. The mature transcript of the cleavage-dependent part is loaded into RISC in complex with the endonuclease Ago2. The processed transcript of the cleavage-independent unit functions as a miRNA by binding to RISC inducing mRNA degradation/P-body sequestration or transcriptional inhibition. In general, the mechanism of bi-shRNAs leads to higher efficacy and greater durability compared with siRNA and miRNA [98].

The current understanding of therapeutic implementation of all these different small RNA approaches has been extensively reviewed: Seyhan discussed the progress and arising challenges in developing RNAi therapeutics against genetic diseases [99]. The current status of RNAi-based anticancer therapy and their potential clinical application is discussed by Wang *et al.* [98] and Bora *et al.* [100].

### 2.4. The Use of RNA Interference against PARP Enzymes

The possibility to suppress the expression of a specific protein in cell cultures has attracted a lot of interest. Moreover, the PAR research was extended to cell systems other than that of murine origin, due to RNAi techniques. Historically the first experiments using RNAi as a tool targeting PAR metabolism were performed aiming the knock down (k.d.) of PARP1 (Table 2).

Already in 2002, Gan *et al.* reported the silencing of PARP1 protein caused by the transfection of PARP1-specific dsRNA molecules in murine neuroblastoma cells [101]. They targeted the N- as well as the C-terminus of the *Parp1* gene, and both approaches diminished the expression of the corresponding protein. Further, this study confirmed the resistance of PARP1 abrogated cells to oxygen–glucose deprivation as a functional consequence. This phenotype was previously demonstrated in cells lacking PARP1 due to genetic ablation. Therefore they suggested the application of RNAi as a powerful tool to study gene functions in cells. In 2004, Kameoka *et al.* introduced RNAi against PARP1 in human cell cultures. In this report, the authors studied the HIV-1 replication within HeLa and J111 cells that have an impaired PARP1 expression [102]. One of the obvious advantages to prefer RNAi rather than chemicals for enzyme inhibition purposes is its specificity. This was demonstrated in an elegant study by Shah *et al.* in 2005 [103]. They developed and compared RNAi approaches targeting the C-terminus of the *parp1* gene in human, murine and hamster cells and investigated the impact of PARP1 k.d. on the expression level of PARP2. As discussed above PARP2 exhibits a significant amino acid and nucleotide sequence similarity with PARP1 in the C-terminal domain. The RNAi target sequences for PARP1 determined in this study have only three (for the murine) or greater than five (hamster, human) mismatches with the *parp2* gene. Shah *et al.* found no impact on PARP2 expression in the tested PARP1 k.d. models as tested by Western blot analyses. They concluded that RNAi against PARP1 does not interfere with its most related protein PARP2 and since other PARP-homologs have even greater disparity to PARP2, it is not likely to affect them by RNAi against PARP1 [103]. To date, RNAi against PARP1 has been applied to at least 20 different human cell types and to cells derived from other mammalian species (Table 2). These include immortalized, primary and cultures from embryonic stem cells.

**Table 2:** RNAi against PARP1 in mammalian cells. Studies labeled with * provide detailed targeting sequence information.

Cell line	Cell type	Species	References
293	Embryonic kidney cells	Human	[105]*, [106,107]
3T3-L1	Preadipocytes	Mouse	[108]*
A20	B-cell lymphoma	Mouse	[109]
A549	Lung adenocarcinoma epithelial	Human	[110,111]*, [107,112,113,114]
AGYNB010	Neuroblastoma	Mouse	[101]*
bEnd.3	Cerebral vascular endothelial	Mouse	[114]
CHO	Ovarian cells	Chinese hamster	[103]*
EW7	Ewing sarcoma	Human	[115]*
GM00637	SV-40 transformed skin fibroblasts	Human	[116]*
H9 hESCs	Embryonic stem cells	Human	[117]*
HaCaT	Keratinocytes	Human	[118,119]
HCT-116	Colon adenocarcinoma	Human	[120,121]
HeLa	Cervix carcinoma	Human	[102,122,123,124,125,126,127,128]*, [129,130]
HepG2	Hepatocytes	Human	[131]
HUVEC	Endothelial cells	Human	[132]
J111	Acute monocytic leukemia	Human	[102]*
Jurkat	T-cell lymphoma (Type II)	Human	[133]*
MCF-7	Breast cancer	Human	[134]*
MEF	Fibroblasts	Mouse	[25,103,135,136]*
NB4	Acute promyelocytic leukemia	Human	[115]*
NIT-1	Insulinoma cells	Mouse	[137]*
PC12	Prostate cancer	Human	[138]
Primary	Fibroblasts	Human	[139]*
Primary	Cerebral cortex neurons	Rat	[140]
Primary	Rheumatoid arthritis synovial cells	Human	[141,142]
Primary	Vascular smooth muscle	Rat	[143]
Ramos	Burkitt’s lymphoma	Human	[109]
SHSY5Y	Neuroblastoma	Human	[144]
SW480	Colorectal adenocarcinoma	Human	[145]
WRL-68	Liver cells	Human	[146]

RNAi molecules targeting PARP2 were next developed to study distinct and overlapping functions of PARP1 and PARP2 in a variety of mammalian cells (Table 3). As described above, PARP2 k.o. has created lethality in conjunction with PARP1 k.o. in mice [70]. Here the application of RNAi techniques helped to overcome this problem. Within the last years we have investigated the combination of either double RNAi (PARP1 and PARP2) in human cells [122,127] or the combination of genetic ablation of *parp1* gene together with RNAi against PARP2 in murine cell cultures [135]. None of the tested systems revealed a lethal phenotype and the cells kept the ability to attach and proliferate under normal culture conditions. Woodhouse *et al.* silenced PARP1 and PARP2 alone or in combination in cell cultures derived from primary human fibroblasts and no toxicity was reported due to double silencing [139]. This putative discrepancy compared to the *Parp1^−/−^* *Parp2^−/−^* mouse models is most likely related to the noncomplete gene disruption mediated by RNAi in all studies [122,135,139,145]. However, here the implication of RNAi provides advantages to study distinct functions of closely related proteins and overcomes lethality problems and the lack of specific inhibitors. Whereas PARP1 and PARP2 are the most studied members of the PARP enzyme family, RNAi was used as a tool to suppress other PARP enzymes including PARP3 [126,147,148], PARP4 (vault-PARP) [126], PARP5A (Tankyrase 1) [126,149], PARP5B (Tankyrase 2) [126], PARP7 (TIPARP) [150] and PARP16 [151].

**Table 3:** RNAi against PARP2 in mammalian cells. Studies labeled with * provide detailed targeting sequence information.

Cell line	Cell type	Species	References
293	Embryonic kidney cells	Human	[72]*, [152]
A549	Lung adenocarcinoma epithelial	Human	[111]
BHK	Baby hamster kidney fibroblast	Chinese hamster	[152]
C2C12	Myoblasts	Mouse	[153]*
HeLa	Cervix carcinoma	Human	[122,126,127]*
MEF	Fibroblasts	Mouse	[135]*
MOVAS	Aortic smooth muscle	Mouse	[154]*
SW480	Colorectal adenocarcinoma	Human	[145]

Moreover, RNAi approaches aiming at the organismic level of invertebrates, mice and plants have been performed to clarify PAR metabolism *in vivo* and/or to investigate a possible clinical application of RNAi itself. Gravel *et al.* published in 2004 RNAi experiments targeting the human tankyrase related gene *pme-5* in the invertebrate *C. elegans* [155]. The same group demonstrated later the effective application of RNAi against the *C. elegans* PARGs pme-3 and pme-4 [156]. The group of Tulin studied PAR metabolism in flies (*Drosophila melanogaster*). They established and investigated RNAi approaches against PARP1 and its counterpart PARG and clarified their involvement in gene expression [157,158]. 

Popoff *et al.* investigated antisense oligonucleotides targeting PARP2 in a colitis mouse model [159]. Recently, Goldberg and co-workers established whole body silencing of PARP1 studying nanoparticle-mediated siRNA delivery in mice [160]. In this analysis, they investigated the tumor dissemination in a Brca1-deficient genetic background after application of siRNAs targeting PARP1. The stress tolerance of PARP1 and PARP2 impaired *Arabidopsis thaliana* as well as oilseed rape (*Brassica napus*) plants have been determined using RNAi tools [161,162]. 

### 2.5. The Use of RNA Interference against PARG

The lack of specific chemical inhibitors and the drastic lethal phenotype of *Parg^−/−^* cells as discussed above evolved into a set of studies using RNAi to suppress PARG in cells (Table 4). We reported previously that silencing of the *parg* gene in murine and human cells lead to an accumulation of PAR after oxidative and alkylating DNA insults [122,163]. The delayed degradation of PAR molecules had a protective effect in particular when cells were challenged with the oxidant hydrogen peroxide (H_2_O_2_). Recently, Feng *et al.* confirmed this finding in human breast cancer cells treated with alkylating agents [164]. Interestingly, the investigation of PARG silencing in mammalian cells after genotoxic challenge has resulted in a hypersensitivity phenotype, as well [111,165,166]. In these studies, cells treated with siRNA targeting PARG forced cell death due to PAR accumulation, similar to results obtained in cells with a genetic PARG impairment [76]. Andrabi *et al.* showed that artificially introduced PAR molecules trigger an apoptosis inducing factor (AIF) mediated cell death pathway [165]. By contrast, an increase of cytoplasmic Ca^2+^ independent from PARP activity has been implicated in the release of AIF from mitochondria subsequently leading to cell death [167]. We followed this hypothesis and found that the inhibition of PARG using RNAi diminishes the occurrence of free ADPR molecules that can trigger a rise in cytosolic Ca^2+^ via the transient receptor potential mediated channels 2 (TRPM2) [25]. As a consequence, AIF translocation—indicative of cell death—was abrogated. To date, this Ca^2+^ channel is the only one activated by monomeric ADPR, and it is dependent on PARP/PARG activity [25,168,169,170,171]. This finding resolves a long lasting controversy about the roles of PAR and ADPR as cell death signals.

**Table 4:** RNAi against PARG in mammalian cells. Studies labeled with * provide detailed targeting sequence information.

Cell line	Cell type	Species	References
16HBE	Bronchial epithelial	Human	[172]*
293	Embryonic kidney cells	Human	[107]
A549	Lung adenocarcinoma epithelial	Human	[111]*, [107]
HeLa	Cervix carcinoma	Human	[122,163,165,166,173]*
LoVo	Colon carcinoma	Human	[174,175]*
MCF-7	Breast cancer	Human	[134,163,164]*, [176]
MEF	Fibroblasts	Mouse	[25,38,163]*
Primary	Rheumatoid arthritis synovial cells	Human	[142]
Primary	Glioblastoma	Human	[177]*
RAW 264.7	Macrophages	Mouse	[178]*

## 3. Conclusions

The implementation of RNAi has markedly expanded our knowledge of the PAR metabolizing system. It has helped to demonstrate distinct functions attributable to PARP members and PARG in a nonmurine background. Moreover RNAi helped overcome lethality problems in mice that were discovered when PARP1 and PARP2 were ablated simultaneously or when the *parg* gene was depleted. The targeting specificity of the RNAi approach is considerably better than chemical inhibitors, which often do not distinguish between individual PARP members or fail to inhibit at all in a cellular context because of bioavailability problems (PARG).
